# Bioprocess and genetic engineering aspects of ascomycin production: a review

**DOI:** 10.1186/s43141-020-00092-0

**Published:** 2020-11-19

**Authors:** Krishika Sambyal, Rahul Vikram Singh

**Affiliations:** 1 University Institute of Biotechnology, Chandigarh University, Gharuan, Punjab India; 2 Academy of Scientific and Innovative Research (AcSIR), Ghaziabad, 201002 India

**Keywords:** *Streptomyces hygroscopicus*, Ascomycin, Immunosuppressant, Fermentation, Yield improvement, Mutagenesis

## Abstract

**Background:**

Ascomycin is a highly valuable multifunctional drug which exhibits numerous biological properties. Being an immunosuppressant, it is known to prevent graft rejection in humans and has potential to treat varying skin ailments. Its derivatives represent a novel class of anti-inflammatory macrolactams. But the biosynthetic machinery of ascomycin is still unclear.

**Main body of the abstract:**

Due to the structural complexity, there occurs difficulty in its chemical synthesis; therefore, microbial production has been preferred by using *Streptomyces hygroscopicus* subsp. *ascomyceticus.* Through several genetic manipulation and mutagenesis techniques, the yield can be increased by several folds without any difficulties. Genetic engineering has played a significant role in understanding the biosynthetic pathway of ascomycin.

**Short conclusion:**

Recently, many efforts have been made to utilize the therapeutic effects of ascomycin and its derivatives. This article covers concepts related to the production kinetics of ascomycin including an update of the ongoing yield improvement techniques as well as screening method of novel strains for ascomycin production.

## Background

**Fig. 1:**
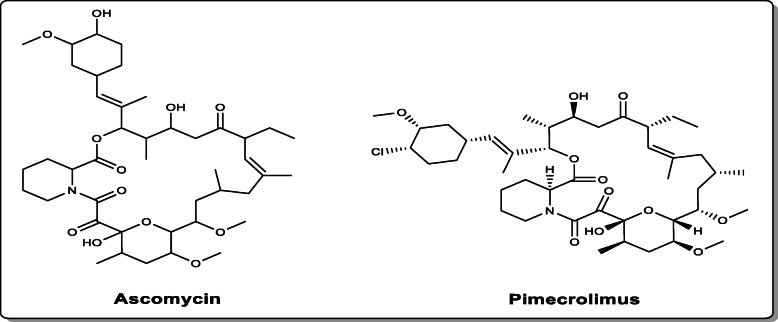
Structures of ascomycin and its derivative pimecrolimus

Ascomycin (FK520) is a pharmacologically significant 23-membered macrocyclic polyketide antibiotic which exhibits numerous biological activities like immunosuppressive [1], anti-malarial [2], anti-fungal, and anti-spasmodic [3] recognized as a highly valuable multifunctional drug [4]. It is used in the medical field for curing many skin ailments. Ascomycin has a structural analog known as tacrolimus (FK506) which is also used as a well-known immunosuppressive agent in the prevention of xenograft rejection after an organ transplant in humans [5]. It has a great potential to enhance regeneration of nerves with high functional recovery [6]. Both ascomycin and tacrolimus exist in the macrophilin-binding region as a mixture of several isomers [7]. Being an important and effective immunosuppressant, ascomycin drug has captivated the clinical industry for the treatment of organ transplant rejections, autoimmune, and skin diseases [3, 8, 9]. Other than an active pharmaceutical component, it is an initiating ingredient for the synthesis of its derivative like pimecrolimus (Fig. 1). This derivative can be an advantageous alternate of ascomycin with enhanced activity due to changes in the side-chain group [4, 10]. It is presently used for first-line treatment of atopic dermatitis (mild-to-moderate) [11]. Psoriasis, seborrheic dermatitis, and vitiligo are some of the skin inflammatory diseases that can also be treated by the therapeutic effects of the derivatives of ascomycin [12–14]. The bacterial class of actinomycetes is known to produce varying biologically active secondary metabolites with *Streptomyces* being the largest actinobacterial genus to form important and valuable therapeutic category of immunosuppressants, anti-cancer compounds, antibiotics, and many more [4, 15]. It is quite difficult to synthesize ascomycin through a chemical route mainly due to its complicated macrolide structure. Other disadvantages include the formation of multi-step, non-eco-friendly reactions, synthesis of side by-products, lower yields, high pH, and temperature conditions. Therefore, microbial fermentation is preferred industrially by using *Streptomyces hygroscopicus* sub sp. *ascomyceticus* [4, 16], though the yield is very low in wild strains as compared to the high production costs. This antibiotic has gained sustained attraction from research laboratories and pharmaceutical industries owing to its multifunctional biological properties as well as market prospects [9]. Recently, many efforts have been made to utilize the therapeutic effects of ascomycin and its derivatives in the medical field for the sake of humans [11, 14]. Therefore, the demands also escalate, and to fulfill them, some considerable efforts such as genetic manipulations via strain engineering and fermentation process optimization are done in the past few years. In regard to the ascomycin production, several attempts are made to overproduce it from available resources. Thus, to enhance the titter value of ascomycin, few strategies explored in strain *S. hygroscopicus* var. *ascomyceticus* include Femtosecond laser irradiation [14, 17], ^13^C-labeling elementary flux mode analysis [18], metabolic profiling analysis [19], use of chemical elicitors [14], ultraviolet mutagenesis [14], PBH as intracellular carbon reservoir [20], role of LAL regulators [21], engineered LTTRs [22], and co-overexpression [20]. There are many more approaches which have great potential and exploration of such novel strategies by biotechnological engineering techniques can help to overcome the limited availability of ascomycin. The aim of the present review is to provide knowledge regarding the biological activities, biosynthesis, optimization conditions, and yield improvement of ascomycin through mutagenesis approach.

## Main text

### Biological activities of ascomycin and its analogs

Ascomycin (FK520) was initially referred to as FR-900520 [7] and isolated from *S. hygroscopicus* KK317 in 1962 [14]. It was due to the high effectiveness of an orally used cyclosporin A that is a cyclic peptide accompanying 11 amino acids [23, 24], which led to the discovery of this novel class of compounds [7]. Tacrolimus (FK506), the analog of ascomycin [9, 11] was first produced by *S. tsukubaensis* in 1984 [25]. After its discovery, another macrolactam was discovered similarly as ascomycin and cyclosporin A in an antifungal screening method of fermentation broths [7, 26] named rapamycin (sirolimus) which is also an immunosuppressant, antifungal, antitumor, and anti-aging drug [7, 27]. Moreover, rapamycin is also useful to prevent restenosis of coronary arteries following angioplasty by acting as a drug eluting stent [24]. It inhibits the growth-promoting cytokine signaling transduction pathway with mTOR instead of calcineurin, thus, interferes with the second phase of T cell activation [28]. These macrolides share a motif that interacts with FK506-binding proteins, resulting in new protein–protein interactions giving rise to many clinically useful activities of these molecules [29, 30]. Besides rapamycin, antascomycin is another ascomycin-related natural product with a,b-diketo-pipecolate subunit [7]. It has been isolated from a strain of *Micromonospora* [31] but its biological properties are yet to be explored. They bind strongly to macrophilin (FK506 binding protein, *Fkb*P12) and antagonize the effects of tacrolimus and ascomycin on T cells [7]. Immunosuppressive drugs can also be called as anti-rejection drugs that make the body less likely to reject a transplanted organ, such as a liver, heart, and kidney [3]. The increasing incidences of chronic ailments requiring the demand for an organ replacement has driven the growth of these drugs in the market. Under submerged aerobic culture conditions, Tadashi et al. reported the production of an anti-fungal agent by cultivation of a strain of *S. hygroscopicus* var. *ascomyceticus*, identified as ascomycin [4, 32]. Filamentous fungi like *Penicillium chrysogenium* can be effectively inhibited by ascomycin at very low concentrations of about one part per million in nutrient agars, whereas many bacterial strains even at a higher concentration cannot be inhibited by it giving an evidence of its anti-fungal property [32]. The anti-inflammatory activity of ascomycin was discovered with reports of treatment of atopic dermatitis, psoriasis, and many other inflammatory skin diseases by high efficacy of oral cyclosporin A which, however, was unable to effectively act as calcineurin inhibitor topically for minimizing systemic immunosuppressive side effects. But tacrolimus and ascomycin type calcineurin inhibitors were seen to be highly effective after topical application when tested on model pigs with ACD. This gave the first pharmacological evidence of the potential of ascomycin and tacrolimus to show anti-inflammation property for treating skin diseases [33]. Intensive studies to identify a compound with high anti-inflammatory activity along with minimal side effects finally resulted in the discovery of pimecrolimus (SDZ ASM 981), an ascomycin macrolactam derivative (Fig. 1). Pimecrolimus has been developed as a new cell-selective inhibitor of inflammatory cytokine secretion with very less adverse effects than presently available drugs. It is known to inhibit an enzyme required for the dephosphorylation of the cytosolic form of NF-AT, thus, preventing the transcription and release of both T-helper type 1 and 2 inflammatory cytokines as well as T cell proliferation [34]. Since the early twenty-first century, tacrolimus ointment and pimecrolimus cream have become the first new class of calcineurin inhibitors, topically, being an alternative to topical corticosteroids for treating atopic dermatitis and other inflammatory skin diseases [7]. However, Zuberbier et al. reported that pimecrolimus is a potent inhibitor of mediator release from dermal mast cells and peripheral blood basophils of humans. It can cause inhibition of anti-IgE induced release of histamine from mast cells and basophils (maximally 73% and 82%, respectively) in a dose-dependent manner (at 500 nmol/L pimecrolimus) strongly; mast cell tryptase (maximally 75%); LTC4 (maximally 32%); and calcium ionophore plus phorbolmyristate acetate-induced mast cell TNF-α release (90% maximum at 100 nmol/L pimecrolimus). Whereas, inhibition with cyclosporin A and dexamethasone was 60% and 28% respectively, during mast cell histamine release. This shows that pimecrolimus, an ascomycin macrolactam derivative can be effective in treating patients with immediate and late-type allergic problems as well as basophil and mast cell-dependent diseases like mastocytosis [35].

These derivatives have a similar mode of action to that of ascomycin but differ in few ways/regards. The use of some immunosuppressants such as tacrolimus and pimecrolimus can increase the risk of malignant cancer in patients [36, 37]. Neuropsychiatric problems such as depression and loss of appetite [38] are the few side effects of tacrolimus while tacrolimus nephrotoxicity results in kidney and liver diseases [39]. Cyclosporin is also equally nephrotoxic and impacts the cardiovascular risk after transplantation [40]. Topical use of tacrolimus ointments has higher permeability as compared to pimecrolimus ointments with lower permeability [41]. But these can cause a burning or itching sensation on the skin [37]. Even though pimecrolimus is similar to tacrolimus in regard to the mode of action but it is more selective and do not effect dendritic cells [42].

### Biosynthesis of ascomycin

**Fig. 2:**
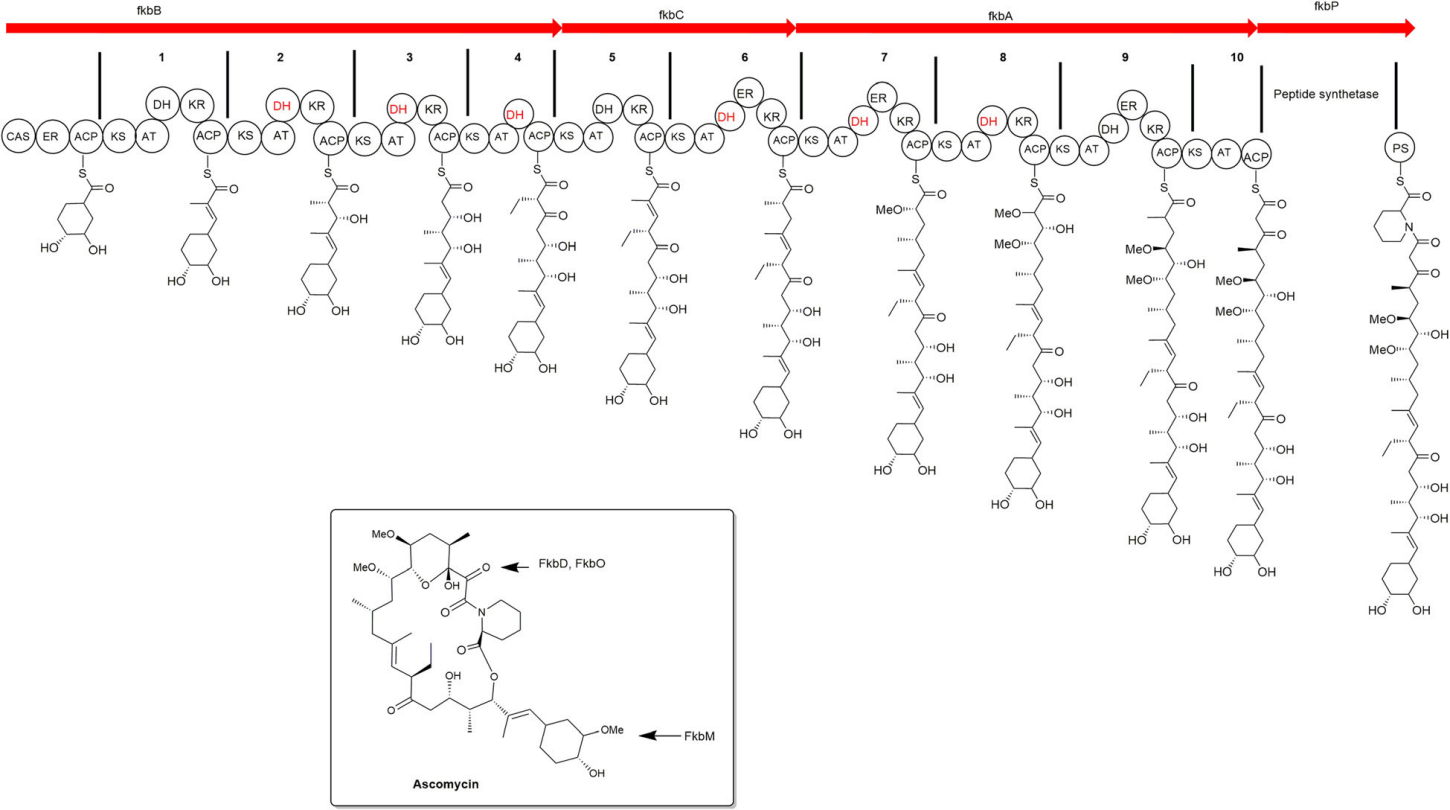
Biosynthesis of FK520 by schematic representation of the PKS complex with circles representing the catalytic domains [25]

In the present time, biosynthesis of ascomycin is totally dependent upon the microbial synthesis due to its structural complexity; therefore, researchers are focusing their attention on microbial synthesis. For the production of ascomycin, strain *S. hygroscopicus* var. *ascomyceticus* is studied well and previous scientific reports concluded that wild strain produced ascomycin in a very little amount; therefore, various yield improvement techniques were applied which proved that there is a large scope of such techniques for yield improvement. Genetic engineering has played a significant role in understanding the biosynthetic pathway of ascomycin [17, 22]. Few studies have been carried out to identify the gene clusters responsible for ascomycin production so that overexpression of such genes in suitable host can improve the yield. Wu et al. studied the overall biosynthesis (Fig. 2) of ascomycin in *S. hygroscopicus* var. *ascomyceticus* (ATCC 14891) and identified four gene clusters (*fkbB*, *fkbC*, *fkbA*, *fkbP*) for biosynthesis of unusual polyketide extender units [25]. Yu et al. studied *S. hygroscopicus* ATCC 14891 for the production of ascomycin and identified seven co-transcription units in the ascomycin biosynthetic gene cluster, including *fkbW*, *fkbU*, *fkbR1/R2*, *fkbE/F/G*, *fkbB/C/L/K/J/I/H*, *fkbO/P/A/D/M*, and *fkbS/Q/N* which were responsible for the synthesis of ascomycin with yield improvement. To determine the expression level, one gene in each of the seven co-transcription units (*fkbW*, *fkbU*, *fkbR1*, *fkbE, fkbB*, *fkbO*, *fkbS*) was selected and results revealed that the expression of four co-transcription units (*fkbW*, *fkbU*, *fkbB/C/L/K/J/I/H*, and *fkbO/P/A/D/M*) were responsible for twofold higher improved yield of ascomycin in *S. hygroscopicus* SFK-36 in comparison to strain ATCC 14891 [11]. Another study was conducted by Wang et al. where three pathways (aminoacyl-tRNA biosynthesis; phenylalanine, tyrosine, and tryptophan biosynthesis; and pentose phosphate pathways) were studied, from which aromatic amino acid and pentose phosphate biosynthesis pathway were seen to be responsible for the synthesis of precursor molecule involved in ascomycin biosynthesis. DHCHC, the precursor of ascomycin and aromatic amino acid biosynthesis are closely related through chorismic acid (via the shikimate biosynthesis pathway); and activation of pentose phosphate pathway also provides more reducing power via NADPH along with erythrose 4-phosphate, a direct precursor of the shikimate pathway important to enhance ascomycin production. As per the earlier reports, to facilitate the overproduction of tacrolimus efficiently, gene manipulation or exogenous feeding was used for the enhancement of the shikimate and pentose phosphate pathway. Therefore, genes from the pentose phosphate pathway (*zwf* gene, encoding glucose-6-phosphate dehydrogenase) and shikimate pathway (*aroA* gene, encoding 3-deoxy-7-phosphoheptulonate synthase) could be selected as potential ascomycin biosynthesis target genes [14]. Zhang et al. studied that the inactivation of *FkbN* led to a complete loss of ascomycin production. A constructed strain FS35/pIBON with *fkbN* overexpression increased the yield by 400% (1800 mg/L), and the expression of genes of encoding precursor, PKS and NRPS were also affected by this regulon. Thus, it is a pathway-specific positive regulator for the biosynthesis of ascomycin. It was also observed that heterologous genetic complementation of *fkbN* deletion strains with a single copy of the homologous LAL family regulators restored the production of ascomycin [21]. Studies suggested that various pathways are available for the production of ascomycin through precursor molecule and it was also observed that every gene cluster is related to each other. But still, it is not clear that how many genes are responsible for the production of ascomycin. Hence, the biosynthetic machinery is not completely known and the key enzymatic steps cannot be located accurately in the ascomycin biosynthetic pathway [17].

### Optimization of fermentation conditions for ascomycin production

Kinetic study of strain plays a very significant role in the production of any metabolite; therefore, determining pattern of growth and product formation approaches of an organism in presence of different nutrient mediums is an important step. Since *S. hygroscopicus* is one of the prominent strains for production of ascomycin at industrial scale [4], few studies have been carried out which show the type of fermentation medium (Table 1) that can be used to achieve a high yield of ascomycin by strain *S. hygroscopicus.* The fermentation parameters like temperature, pH, and agitation must be optimized initially to design an efficacious production media providing with an improved metabolite production in the future. Identification of appropriate medium components and optimization of production parameters in shake flasks is necessary before carrying out scale-up processes [15]. For preparation of the spore stocks of *S. hygroscopicus* ATCC 14891, Reeves et al. used SY medium for the production of ascomycin and its analogs in tryptic soy broth with 50 mM TES buffer, pH 7, and 1% glucose (TSBGM) [5]. Qi et al. used *S. hygroscopicus* var. *ascomyceticus* FS35 as a starting strain for obtaining a mutant strain *S. hygroscopicus* SA68 from combination of femtosecond laser irradiation and shikimic acid enduring screening which resulted in 270 mg/L ascomycin. The seed and production medium were prepared in an unbaffled shake flask [9] (Table 1). *Streptomyces hygroscopicus* var. *ascomyceticus* FS35 was studied by Song et al. for the production of ascomycin in fermentation medium (Table 1). They explored the role of the LTTR FkbR1 in ascomycin biosynthesis and used the soluble fermentation medium to culture mycelia for RNA isolation and ChIP assays [22]. To maximize the growth of *S. hygroscopicus* sub sp. *ascomyceticus* ATCC 14891 for increasing ascomycin production, Luthra et al. optimized media components (Table 1) by using shake flasks for future scale-up studies. Two sets of experiments were carried out, namely, two-stage fermentation and three-stage fermentation. It was observed that in two-stage fermentation with transfer of lab-grown inoculum directly into production media, the ascomycin production was maximum in P3 and P8 mediums i.e., 0.216 mg/g and 0.172 mg/g respectively on the 11th day of incubation at 28 °C while in three-stage fermentation with transfer of lab-grown inoculum initially into seed media followed by transfer of seed media to production media, the production was increased by addition of a seed-stage S2, giving 0.296 mg/g ascomycin in P3 and 0.356 mg/g in P8 medium [4]. A high-yield *S. hygroscopicus* SFK-36 strain was obtained by Yu et al. through ARTP-induced mutagenesis of the original strain *S. hygroscopicus* ATCC 14891. After ARTP-induced mutagenesis of the original strain, mutant *S. hygroscopicus* SFK-36 was able to produce 495.3 mg/L of ascomycin but the production medium was further optimized by RSM to enhance the production up to 1466.3 mg/L of ascomycin in flask level. SFK-36 showed a faster carbon utilization rate in the optimized medium (Table 1). Furthermore, production was carried out at 5 L fermenter and after 192 h, 1476.9 mg/L of ascomycin was recovered. Results concluded that combination of traditional mutagenesis breeding and medium optimization can be an effective approach for enhancing ascomycin production [11]. Another study, the effect of chemical elicitors on ascomycin production was carried out by Wang et al. using *S. hygroscopicus* var. *ascomyceticus* H16 strain cultured in suitable medium (Table 1). Sterile chemical elicitors were also added separately in the production mediums where a yield of 182.15 ± 11.86 mg/L was achieved in medium with 0.6% DMSO at 96 h that continued increasing till 168 h [14]. Ascomycin is an intracellular metabolite which is present in cell pellets; therefore, complete extraction of product from cell biomass is also a crucial step in the whole process. In most of the studies, ascomycin production was done in shake flask to batch fermenter for 3 to 5 days to complete the process except for *S. hygroscopicus* subsp. *ascomyceticus*, ATCC 14891 which took an operation time of 10-14 days [4]. Few kinetics regarding the strain *S. hygroscopicus* are also given in Table 1.

**Table 1:** Media components and fermentation conditions for ascomycin production

S. No	Microorganism	Media components	Bioreactor	Operate mode	Time (h)	Reference
1	*S. hygroscopicus* ATCC 14891	**Growth medium:** TSBGM (tryptic soy broth with 50 mM TES buffer pH 7 and 1% glucose)	**Baffled shake flask**	**Fed batch**	**56**	[5]
2	*S. hygroscopicus* var. *ascomyceticus* FS35	**Seed medium:** 10 g/L soluble starch, 30 g/L glucose, 6 g/L peptone, 6 g/L yeast powder, and 2 g/L CaCO_3_. **Production medium:** 24 g/L soluble starch, 40 g/L dextrin, 5.0 g/L peptone, 7.0 g/L yeast powder, 2.0 g/L corn steep liquor, 11 mL/L soybean oil, 0.5 g/L K_2_HPO_4_·3H_2_O, 1.5 g/L (NH_4_)_2_SO_4_, 1.0 g/L MgSO_4_·7H_2_O, and 1.0 g/L CaCO_3_. **pH**: 6.7	**Unbaffled shake flask**	**Batch**	**168**	[9]
3	*S. hygroscopicus* var. *ascomyceticus* FS35	**Production medium:** 20 g/L soluble starch, 40 g/L dextrin, 5 g/L yeast powder, 5 g/L peptone, 5 g/L corn steep liquor, 1 g/L K_2_HPO_4_, 1.5 g/L (NH_4_)_2_SO_4_, 0.5 g/L MnSO_4_, 1 g/L MgSO_4_·7H_2_O, 1 g/L CaCO_3_, and 2.5 mL/L soybean oil. **Soluble medium for RNA isolation and ChIP assays:** 30 g/L glucose, 2 g/L (NH_4_)_2_SO_4_, 1 g/L NaCl, 1 g/L K_2_HPO_4_, 1 g/L MgSO_4_·7H_2_O, 0.001 g/L FeSO_4_·7H_2_O, 0.001 g/L MnCl_2_·7H_2_O, 0.001 g/L ZnSO_4_·7H_2_O, and 10 g/L MOPS.	**Shake flask**	**Batch**	**168**	[19]
4	*S. hygroscopicus* subsp. *ascomyceticus* ATCC 14891	**Growth medium:** (YMA) 4.0 g/L yeast extract, 10 g/L malt extract, 4 g/L glucose, and 15 g/L agar. **pH**: 6.5–7.0	**Shake flask**	**Batch**	**240-336**	[4]
5	*S. hygroscopicus* ATCC 14891	**Seed medium:** 8.0 g/L corn steep liquor, 10.0 g/L glucose, 3.0 g/L cottonseed meal, and 1.0 g/L KH_2_PO_4_. **pH:** 7.0 **Production medium:** 20.0 g/L soluble starch, 40.0 g/L dextrin, 5.0 g/L yeast powder, 5.0 g/L peptone, 5.0 g/L corn steep liquor, 1.0 g/L K_2_HPO_4_·3H_2_O, 1.5 g/L (NH_4_)_2_SO_4_, 0.5 g/L MnSO_4_·H_2_O, 1 g/L MgSO_4_·7H_2_O, 1 g/L CaCO_3_, and 1 g/L soybean oil. **pH**: 6.5	**Shake flask**	**Batch**	**168**	[11]
6	*S. hygroscopicus* SFK-36	**Production medium:** 81.0 g/L soluble starch, 57.4 g/L peanut meal, 15.8 g/L soybean oil, 0.5 g/L MnSO_4_·H_2_O, 1 g/L K_2_HPO_4_·3H_2_O, 1 g/L MgSO_4_·7H_2_O, and 1 g/L CaCO_3_. **pH**: 6.0-7.0.	**5 L fermenter**	**Batch**	**192**	[11]
7	*S. hygroscopicus* var. *ascomyceticu*s H16	ISP4 medium including synthetic and complex medium. Filtered chemical elicitors added to production medium.	**Shake flask**	**Batch**	**192**	[14]

### Mutagenesis approach for the yield improvement

**Scheme 1:**
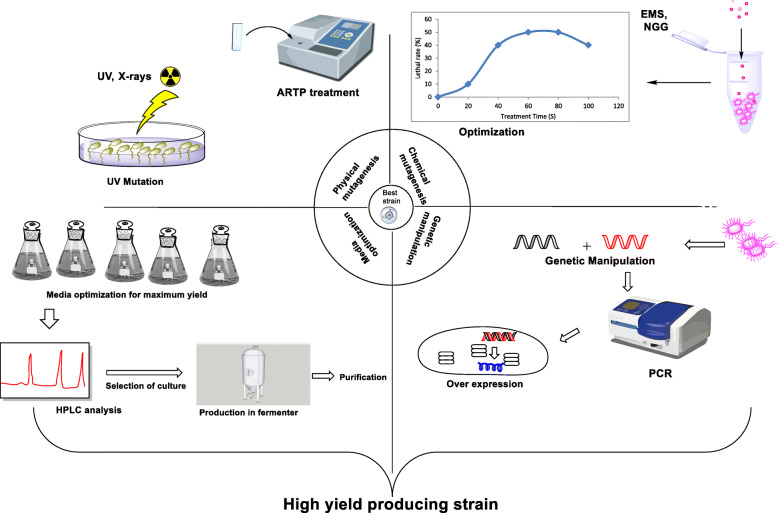
Different strain improvement approaches used for production of ascomycin to get higher yield

Ascomycin is a multifunctional drug but due to its complex macrolide structure, there is difficulty in the chemical synthesis providing a lower yield, limiting its large-scale commercial production. Therefore, the production through microbial fermentation is of great significance [16]. Even though the yield via this process is also less with high production costs, thus, numerous engineering approaches have been made to achieve higher ascomycin yields (Table 2). Tadashi et al. studied the production of ascomycin with varying culture conditions. Under submerged fermentation, strain *S. hygroscopicus* no. KK317 was grown aerobically for 12 h supplemented with aqueous carbohydrate solution and nitrogenous nutrients. After extraction, 200 mg/L of a flocculent white precipitate of purified ascomycin was achieved. To improve its yield, several methods were carried out (Scheme 1) [32]. Qi et al. used femtosecond laser irradiation technology for *S. hygroscopicus* at the optimal irradiation conditions of 25 mW for 6 min. After media optimization, a maximum yield of 300 mg/L ascomycin was achieved [17]. It was seen that exogenous oils, shikimic acid, and lysine promoted ascomycin production but the strain *S. hygroscopicus* no. KK317 was more sensitive to shikimic acid. Thus, a mutant *S. hygroscopicus* var. *ascomyceticus* SA68 was prepared by Qi et al. which showed resistance to shikimic acid yielding 330 mg/L ascomycin. The supplementation of this strain with 3 g/L shikimic acid at 24 h resulted in 33% yield increase, i.e., from 330 to 450 mg/L indicating fermentation production of ascomycin through this process at a large scale [9]. Another different strategy used by Qi and co-workers includes addition of adsorbent resin HP20 in the production medium with *n*-hexadecane, valine, and lysine which promoted 53.3% higher ascomycin yield as compared to the initial conditions. This attempt revealed that a combinatorial approach by addition of resin HP20 with rational supplements of valine and lysine in the fermentation can eventually improve the yield of this macrolide [19]. Qi et al. identified two targets with potential (*pyc* and *fkbO*) for gene manipulation and engineered them to form *S. hygroscopicus* TD-ΔPyc-FkbO strain from SA68 (a stable-high yield strain), after integration of ^13^C-labeling experiments and elementary flux mode analysis which resulted in an increased ascomycin yield of 610 mg/L [18]. Wang et al. identified two target genes namely *ccr* and *hcd* which, after simultaneous over-expression resulted in 1.43-fold improvement producing 438.95 mg/L of ascomycin. This was possible by construction of the genome-scale metabolic network model of *S. hygroscopicus* var. *ascomyceticus* which allowed the identification of *ccr* and *hcd* [43]. Song et al. identified the role of LTTR *FkbR1* in ascomycin synthesis, and RT-PCR analysis was done to speculate the potential genes regulated by *fkbR1* and confirmed by in vitro EMSAs and ChIP-qPCR assay. *FkbR1* and its target gene *fkbE* were engineered, and an over-expression strain *OfkbRE* was constructed with combination of both *fkbR1* and *fkbE*. It showed 69.9% of yield increment, i.e., up to 536.7 mg/L ascomycin when compared with that of the parent strain *S. hygroscopicus* var. *ascomyceticus* FS35 providing an evidence of *FkbR1* being a pathway-specific activator for ascomycin biosynthesis [22]. Enhancement of copy numbers of antibiotic biosynthesis gene clusters [44], manipulation of regulatory genes [45], genome shuffling [46], and modification of the transcription and translation machinery [47] are some of the reported methods for overproduction of antibiotics. Chemical elicitors are being used nowadays to accumulate secondary metabolites.

**Table 2:** Different strain improvement strategies employed in the production of ascomycin

S. No	Microorganism	Techniques used	Yield (mg/L)	Reference
1	*S. hygroscopicus* var. *ascomyceticus* FS35	Femtosecond laser irradiation technology	300	[17]
2	*S. hygroscopicus* var. *ascomyceticus* SA68	Shikimic acid resistance and femtosecond laser mutation	450	[9]
3	*S. hygroscopicus* TD-ΔPyc-FkbO	Gene manipulation of *pyc* and *fkbO* target genes	610	[18]
4	*S. hygroscopicus* var. *ascomyceticus*	Over-expression of *ccr* and *hcd* target genes	438.95	[33]
5	*S. hygroscopicus* OfkbRE	Engineered LTTR *FkbR1*	536.7	[19]
6	*S. hygroscopicus* var. *ascomyceticus* H16	Chemical elicitation and combined over-expression of *aroA*, *fkbN*, and *luxR*/ultraviolet mutagenesis and femtosecond laser irradiation.	1258.30 ± 33.49	[14]
7	*S. hygroscopicus* SFK-36	ARTP mutagenesis	495.3	[11]
8	*S. hygroscopicus* SFK-36	RSM	1476.9	[11]

As mentioned above, the successful metabolic engineering strategies providing higher ascomycin yields include reconstruction of *S. hygroscopicus* var. *ascomyceticus* SA68 using (13)C-metabolic flux analysis [18], engineering strains by gene inactivation, and overexpression [14, 43]. The construction of genome-scale metabolic models (GSMMs) of *Streptomycetes* sp. can also be done to describe metabolic pathways and predict favorable routes for the production of secondary metabolites as done by Wang and co-workers for tacrolimus. After GSMM of *S. tsukubaensis*, an overexpression of *aroC* and *dapA* genes produced 1.64-folds higher yield of tacrolimus as compared to the wild-type strain [48]*.* A similar approach can be followed for ascomycin as well. Wang et al. investigated the impact of some chemical elicitors on ascomycin production in *S. hygroscopicus* var. *ascomyceticus* and found that the highest yield was achieved with DMSO treatment. Metabolomics analysis was carried out to identify potential ascomycin biosynthesis target genes and engineered to construct desirable over-expressed strains, all of which showed an improved yield. But the combined over-expression of aroA, fkbN, and luxR genes gave a maximum of 4.12-fold higher yield achieving 1258.30 ± 33.49 mg/L ascomycin production [14]. For improvement in ascomycin production, Yu et al. treated the parent strain *S. hygroscopicus* ATCC 14891 with ARTP mutagenesis to obtain a stable high ascomycin producing *S. hygroscopicus* SFK-36 strain which produced 495.3 mg/L ascomycin in the optimum fermentation medium conditions designed by RSM [11]. Recently, Wang and co-workers reported that the use of PHB as an intracellular carbon source in medium provides a significant increased yield of 2.11-folds than the parent strain. It works as an intracellular reservoir of carbon stored as polymers when carbon sources are abundant while depolymerizing to monomers for the biosynthesis of precursors when the carbon sources are not sufficient. Therefore, the enhancement of PHB metabolism can be marked as a new strategy for a high yield of secondary metabolites [20].

### Future prospects

**Scheme 2:**
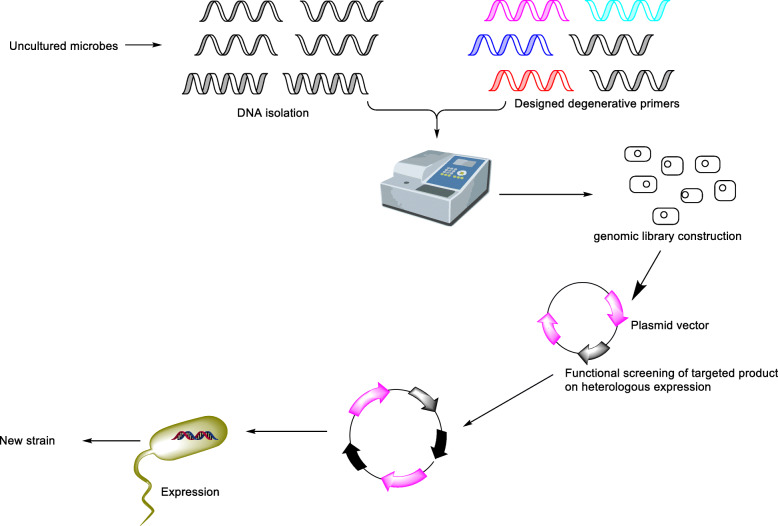
Genome mining approach for ascomycin producing microbes

Traditional isolation and screening methods of microbes for natural products are laborious and time consuming; therefore, scientists are focussing on new and rapid screening techniques from which genome mining (Scheme 2) is one of the effective methods. Most of the microbes are still uncultured; therefore, there is a need to develop rapid screening techniques like HTS, and genome mining, and genome mining can play an extraordinary role in search of such natural products [49]. It is a major challenging step but much of this tedious time-consuming process nowadays can be done in silico by identifying the conserved regions in the genomes of the producer microbes. Since there is an increment in the number of whole-genome sequences of natural products-producing microbes, the abundance of secondary metabolite producing biosynthetic gene clusters will also increase and be identified through the genome mining approach. It helps to identify clusters in genomic data to know about the corresponding chemical molecules, which is now widely used over the classical screening methods [50].

The desired ascomycin-producing genes can be identified from the extracted DNA taken from environment, and its sequence homology can be performed using designed motifs (degenerate primers) which bind only to highly conserved regions. Computational analysis can play an important role in this type of screening technique and provide with biosynthetic gene clusters. Further, whole experiments can be executed in in vitro conditions. After isolation of the desired DNA sequence, it is inserted into a suitable vector (plasmid, cosmid, bacterial artificial chromosome, etc.) and expressed into a suitable host to form a new strain with high ascomycin yielding capacity. Genome mining has a bright future with some striking advantages, viz., it is fast, time saving; cheap and large amounts of data are publically available online. On the other hand, cloning and overexpression of available resources of microbial strains at international/national repositories have the potential to improve the yield by expressing the gene cluster into a suitable host system. Therefore, construction of genomic library is necessary for particular gene clusters involved in production followed by their overexpression in the host system. Apart from all yield improvement approaches considered initially, the production kinetics study of recombinant strain will help to increase the titter value of ascomycin.

## Conclusions

Ascomycin exhibits a potent pharmacological importance; therefore, its demand has increased; however, available strains are producing it in limited amounts. This gains the attention of researchers in yield improvement for such strains by using biotechnological applications. Present review has been framed to give a perspective that biotechnology engineering has the potential to address this issue at present time. Genetic manipulation strategies are being employed to improve the strain yields by gene alteration (either temporary or permanent) for ascomycin production by enrichment with effectiveness. The efforts including media optimization, use of epigenetic modifiers, and chemical/biological elicitors have already been widely exploited in the past decades. Currently, the chemical mutagens, use of PHB, GSMMs, metabolic flux analysis, engineering strains by gene inactivation, overexpression, and precursors for ascomycin production are in trend. Synthetic biology can play an important role for improvising its production in association with those precursors, hence, enhancing the ascomycin yield. Thus, the development of both theories and technologies in regard to novel systems biology can be a promising aid in designing of high yield producing strains. Acceptance of new trends in the production of ascomycin will be a milestone; therefore, more exploration of gene clusters is needed to fulfill the rising macrolide demands. These strategies will permit a better understanding of the biosynthesis pathway and efficient overproduction.

## Data Availability

Not applicable

## Abbreviations

LTTR: LysR-type transcriptional regulators
mTOR: Mammalian target of rapamycin
ACD: Allergic contact dermatitis
NF-AT: Nuclear factor of activated T cells
LTC4: Leucotriene C4
DHCHC: (4R, 5R)-4,5-dihydroxycyclohex-1-enecarboxylic acid
NADPH: Nicotinamide adenine dinucleotide phosphate
ChIP: Chromatin immuno precipitation
RSM: Response surface methodology
RT-PCR: Reverse transcription-polymerase chain reaction
ChIP-qPCR: Chromatin immuno precipitation-qPCR
EMSAs: Electrophoretic mobility shift assays
DMSO: Dimethyl sulfoxide
ARTP: Atmospheric and room temperature plasma
PKS: Polyketide synthases
NRPS: Nonribosomal peptide synthetaseS
LAL: Large ATP-binding regulators of the LuxR family
WGCNA: Weighted correlation network analysis
GSMM: Genome-scale metabolic models
PHB: Polyhydroxybutyrate
HTS: High throughput screening
